# Stem cells and oral surgery: A systematic review

**DOI:** 10.4317/jced.56571

**Published:** 2019-12-01

**Authors:** Regina Mosquera-Perez, Ana Fernández-Olavarria, Rosa-Maria Diaz-Sanchez, José-Luis Gutierrez-Perez, María-Ángeles Serrera-Figallo, Daniel Torres-Lagares

**Affiliations:** 1 DDS. Department of Stomatology, Faculty of Dentistry, University of Seville (US), Seville, Spain; 2 MD, PhD. Department of Stomatology, Faculty of Dentistry, University of Seville (US), Seville, Spain; 3 DDS, PhD. Department of Stomatology, Faculty of Dentistry, University of Seville (US), Seville, Spain

## Abstract

**Background:**

Considering the structural loss that occurs after surgical procedures for cystic and tumoral pathology, in periodontitis, as well as the maxillary atrophy that determines the rehabilitation with dental implants, it is imperative to find satisfactory solutions. The opportunity provided by the findings in stem cells is a recent introduction in the field of oral surgery, based on the regenerative potential that these cells possess in order to restore defects at different levels of the oral cavity. The aim of this systematic review is to discover the real applications that stem cells may have in our treatments in the near future.

**Material and Methods:**

We made a systematic review of the literature on the subject of stem cells to know the publications relating to them in the field of oral surgery since 2000. PRISMA statement was accomplished, as its official flow chart is used.

**Results:**

This article draws clinical conclusions from basic research and those conducted in the first clinical cases to apply them in a short period of time to our patients in order to achieve excellence in regenerative therapies.

**Conclusions:**

To summarize, stem cells may be a turning point in tissue regeneration, though the major challenge is to overcome the remaining obstacles before they become a realistic therapeutic alternative.

** Key words:**Stem cells, oral surgery, cell therapy, regeneration.

## Introduction

When embryonic stem cells were first identified and isolated in mice, it meant a milestone in the history of science (1,2). Before, in the 90s of last century, embryonic stem cells had been obtained from primates. In 1998 the researcher Thomson succeeded for the first time in isolating a lineage of embryonic stem cells, obtained from the interior of the cellular mass of an embryo in a 4-7 days blastocyst (3).

**Figure 1:**
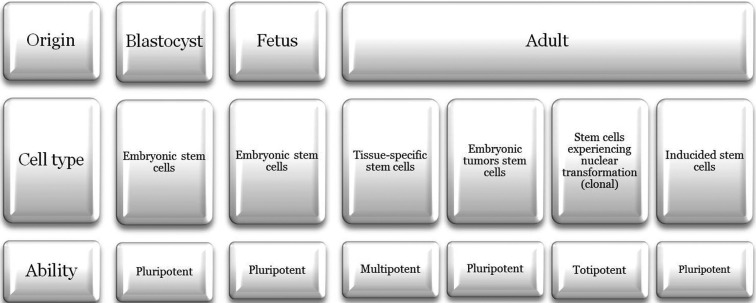
Classification of types of human stem cells, according to its origin, cell type and the degree of differentiation that can develop.

First, we consider it necessary to clarify the fundamental terms used in this systematic review. Stem cells can be defined as the cells responsible for of the foundation of each and every organ and tissue of in the human body. They have two defining characteristics (4): ability to renew themselves indefinitely, producing new stem cells, as well as the ability to differentiate, at the same time, into specialized daughter cells which can perform specific functions. These two features are governed by extracellular signals coupled to intracellular signaling cascades. The information that specifies the fate of stem cells is encoded by the presence or absence of these signals, and also for their combination, location, level and timing. In general, stem cells can be divided into two subtypes: pluripotent and multipotent. The former, also known as totipotent, can differentiate into any type of cell in the human body. They are also stem cells those that are called multipotencial, which can differentiate into many different cell types within their lineage. In reference to the origin of stem cells, they can be obtained from different sources: from the interior of the mass of cells of the blastocyst, prior to the implantation of the embryo, together with fetal and adult tissue. To date, six types of cells have been isolated mother (5-7) as is summarized in Figure 1. Found in most human tissues, they are delivered from tissues undergoing constant self-renovation, as well as other tissues with a lower renewal capacity. Being designed to work in tissues in the long run, adult stem cells are responsible for their maintenance and repair by replacing cells which are damaged or have been lost. Considering that adult stem cells are generally multipotent, they may form a limited number of cell types, corresponding to their tissues of origin.

**Figure 2:**
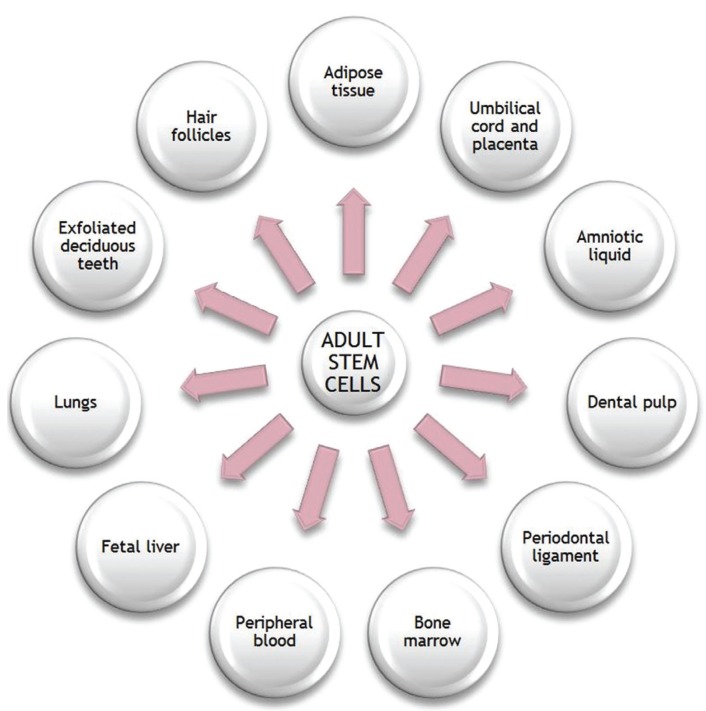
Different tissues where the existence of adult stem cells have been shown.

 The presence of adult stem cells (Fig. 2) has been found in various tissues. Apart from the bone marrow, which is an important source of mesenchymal stem cells, the detection of adult stem cells in the oral cavity is notable, being present in exfoliated deciduous teeth, dental pulp and periodontal ligament. These cells represent a population of post-natal stem cells capable of performing an extensive proliferation with a multipotent capacity for differentiation (8). Adult stem cells that reside in the mucosa of the oral cavity have a dual capacity, both osteogenic and neurogenic. Nevertheless, these cell populations of the oral cavity are not completely characterized, few stem cells have been isolated so far, and their markers are still unknown.

Tissue engineering requires the selection of an ideal source of stem cells; for which they need to allow several conditions (9), including the easy access and the availability of the donor area, a large capacity of self-renewal or expansion in the place of origin, as well as an ability to easily differentiate into cell lineages of interest issued by the signaling molecules. In addition, it is essential to prove an absence or minimal immunogenicity as well as a minimal risk of malignant transformation capacity. Referring to the donor site of stem cells, they are usually cultivated from iliac crest bone marrow candidates, although the extraction procedure can be painful and laborious. It is interesting that mesenchymal stem cells can be obtained from the periosteum of the mandible, accessible to the oral surgeon under local anesthesia, obtaining mesenchymal stem cells of the periosteum with a proven osteogenic potential. In addition, it can be obtained from the maxillary tuberosity. Samples can be cultivated *in vivo* during a certain period of time, scattered in biomaterial that serves to give structure to the cells, and then transplanted into the defect we desire to regenerate, as in a sinus lift procedure.

There are products available based on cells obtained from bone marrow mesenchymal stem. These products contain stem cells suitable to differentiate into various cell types, and can respond to the medium where they are inserted to differentiate themselves in the appropriate tissues, as needed. Moreover, these products can be provided to patients without the inconvenience and potential complications from obtaining an autologous graft.

Dental practitioners have to deal on many occasions with patients´ structure loss in different levels of the oral cavity: tooth, periodontal tissue, alveolar bone and soft tissue. Scientific investigations have been forced to try to find more effective methods to the traditionally used to replace lost tissues. In addition to reaching the best levels of health and functionality of the oral cavity, the dentist is subject to the patients´ needs, primarily seeking the best aesthetic result conditioned by economic aspects.

In this article we will focus on progress in issues of regenerative therapies that dentists can use in the field of oral surgery. The problem that arises is the need to find more satisfactory solutions to the loss of structures, as occurs after surgeries of cystic and tumor pathologies, in maxillary atrophy of the patient partially or totally edentulous, implantology treatments, grafts, etc.

Our main goal is to update on this matter that we consider promising, and which from our point of view, dentists are not informed enough in order to make these therapies known to their patients. The possibility of access to rigorous bibliographic sources gives us the opportunity to learn about everything concerning stem cells, which is presented as an interesting alternative to the multitude of regenerative therapies of doubtful predictability and unconvincing results known up to now in oral surgery and periodontics.

## Material and Methods

The first step for this systematic review of the literature on the subject of stem cells was to know the publications relating to them in the field of oral surgery. PRISMA statement was accomplished, as its official flow chart is used. The method used to obtain this information was to perform a search of articles in the database PubMed. Our search strategy selected the key words “Oral Surgical procedures” (Mesh) AND “Stem Cells” (Mesh). More results were obtained including the MeSH terms. The eligibility criteria consisted in choosing all the studies published since 2000, as from that date on it a large increase in the production of scientific publications in relation to the topic of our search can be observed. Also, we excluded articles without English translation and those only published on books. In addition to the main bibliographical search, other searches of additional publications (extracted from the reference lists of the articles found in the main search in PubMed) have been carried out for this paper, as books and articles that did not meet the inclusion criteria.

Then, a full analysis of the texts was conducted. The reason we did not focus on strictly analyzing those studies performed on patients and clinical studies, taking into account that the ultimate aim of this work is to find possible applications of stem cell therapy for our patients, is that we consider that there are not sufficient studies on humans with powerful statistical evidence; since most of the published studies are in the experimental phase or are based on clinical cases performed on few patients. This should be taken into account because they have a design that does not allow extrapolation to the general population, and in many cases do not provide statistically significant results in humans.

For this reason, it was imperative to obtain a wide range of studies, both in vitro and in vivo: research, in experiments on animals, as well as studies on patients, regardless of the number of subjects tested, and if they used control groups or not. Despite being also included, this does not mean that all of these features have not been taken into account. On the contrary, these data were selected from each of the articles to make a clear statement of the results obtained in our search. They were roughly two types of articles: primary research articles and reviews of the literature.

## Results

**Figure 3:**
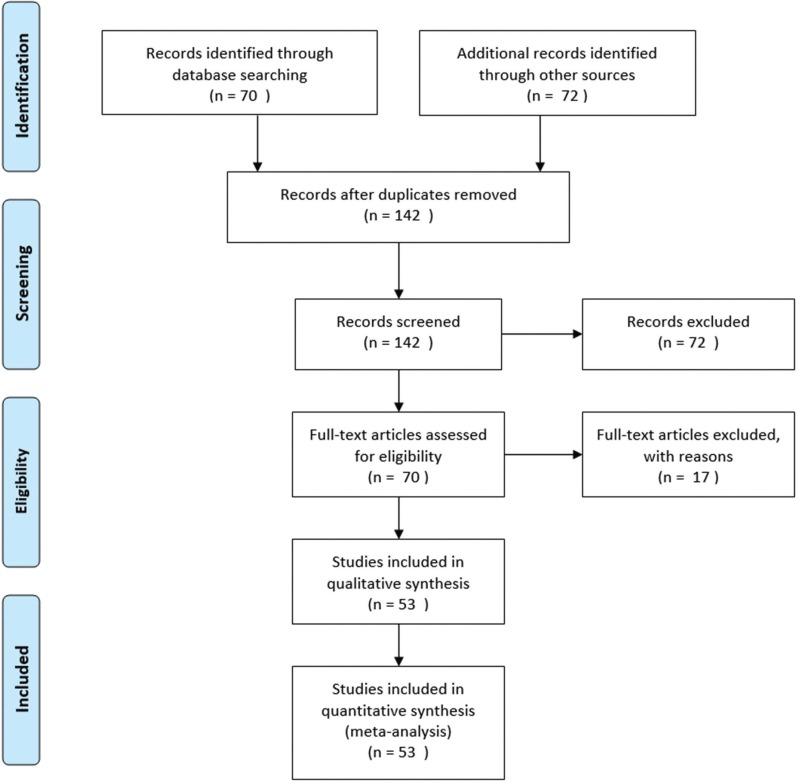
Flow diagram. Study selection.

Figure 3 shows the study selection on a flow diagram, with the selection of studies from the search strategy. In addition, it reveals how 72 articles from the additional search were excluded for the principal analysis, as well as 17 articles that were excluded after applying exclusion criteria. Studies included in the qualitative synthesis were 53. Of these 53 selected articles, 19 were reviews of the literature and were research articles. Of the group of primary research articles, 23 studies have been conducted on animals. This necessary step prior to its application in humans, implies a lower stage on the pyramid of scientific evidence compared to studies on patients. Our research included an article (10) carried out with cells of embryonic origin. Regarding studies performed on patients, there were a total of 10 articles. Furthermore, all of them used a very small population sample: 2 patients (11), 11 patients (12), 1 patient (13), 3 patients (14), 7 patients (15), 5 patients (16), 7 patients (17), 3 patients (18), 3 patients (19) and 3 patients (20).

## Discussion

Will these materials work on our patients?

The findings of the different studies draw an affirmative answer. However, there are still some considerations that prevent stem cells from being an adaptable therapy for our patients. For instance, in most of the studies multiple biomaterials are mentioned to be used along with the stem cells. It is known that its utility in the majority of cases is to act as a filler”, recovering the architecture of the receiving area, without meaning that they are able to cause regeneration of the tissues. All studies agree that histological evidence is the only way to confirm that regeneration has taken place, therefore it is necessary to carry out further studies on patients (1-5,9,19).

What are the basic pillars of the cell therapy?

This can be summed up in the triad of the tissue engineering of Nakashima et al (21), which is divided into three basic components of biological tissues: the response of the cells, an extracellular matrix that serves as scaffolding and induction of morphogenetic signals, as the BMPs. This triad is essential for the regeneration of the oral cavity´s structures. For example, during the process of healing with calcium hydroxide paste, the cells that respond are the pulp cells that migrate and morphogenetic signals are factors of growth, differentiation and BMPs, that have been stored in the dentin matrix and released at the site of the lesion. In this case oesteodentin is the matrix where cells may adhere.

In which surgical procedures could regeneration using stem cells be used?

Some of the surgical procedures mentioned in the articles of our main search, both reviews and research articles, are:

• Periodontal regeneration (14,22-31) 

• Reconstruction of large bone defects (27,32-33) 

• After respective surgeries of cysts and tumors (13)

• Periapical surgery (34)

• Caused by osteoradionecrosis (35)

• Mandibular distraction (36-37)

• Surgical treatment prior to implants

• Sinus lift (9,12,38)

• Increase of crestal bone and to improve the osseointegration (11,18,39-40)

Also, it has been shown that adult stem cells play a key role in the healing of the post extraction sockets (31,41). Despite not being our revision´s issue, there are several non-surgical procedures where the use of stem cells is proposed, as in dentin regeneration (21,31). Its use is also being fully studied in endodontic therapy, as mentioned in a publication (25).

What methods do we have to apply stem cells in oral surgery?

There are almost as many methods of applying stem cells as studies have been published. This occurs because there are still no protocols established regarding biomaterials that should be used for their conduction. Therefore, every research group proposes its method based on previous studies or other publications.

The creation of bone graft from ectopic production is frequently mentioned, for instance, to expanse and differentiate stem cells *in vitro*, and subsequently, when the appropriate analysis considers that there is a good osteogenic activity, said graft is transplanted to the area demanding regeneration. Something similar happens with the stem cells obtained from blood extraction procedures (11) that can be used in treatments prior to rehabilitation with dental implants, as in sinus lift (38).

A mixture of autologous stem cells with all kinds of bone grafts is also common, as already described in the introduction and that the oral surgeon is more than familiar with. They can be used with graft, animal or synthetic origin (23) or autologous (43-44). The use of a fibrin gel like material that eases its application is illustrated, as in the case of injectable bone (39). The use of an injectable preparation consisting of adult autologous stem cells combined with platelet-rich plasma gel is also documented in a study on dogs (33). Another study which suggests the use of platelet-rich plasma is published by Ito K et al (40).

The use of biocompatible membranes, especially in the case of periapical surgery is a controversial subject. Autologous grafts seem to achieve better results without collagen membranes (44). On the other hand, the simple application of a membrane with or without bone graft for periapical surgery, may not result in a regeneration of periapical tissues (34). This is due to the fact that these biomaterials are not able to perform the recruitment of stem cells or signaling molecules that induce mesenchymal cells to differentiate into pre-osteoblasts, periodontal ligament, and pre-cementoblasts forming cells. The beginning of the healing of the periapical lesion after periapical surgery is similar to what occurs in a wound in the connective tissue in any other part of the body (34-35). Concerning that this healing ideally means not only regeneration of alveolar bone, but also of the periodontal ligament and cement after periapical surgery, stem cells might achieve complete periapical tissue regeneration, as an ideal result of periapical surgery. Therefore, as in the post-extraction sockets, the existence of a blood clot from the host provides an excellent natural scaffold for proper healing of surgical wounds (20,34,41). The same happens in studies of mandibular distraction (36-37), where the action of stem cells is stimulated due to surgical technique, or orthopedics in the treatment of mandibular advancement (37).

There are several materials that are not so frequently used in oral surgery but mentioned in certain research studies, as β-TCP (46-47), the gelatin chitosan (41), poly-lactic-glycolic acid (19) or hydroxyapatite (35). These materials are mostly supplemented with grafts, and there are studies in which the authors compare some materials in study groups to check the differences among them.

Does the advanced age of the patients affect to the response to treatment with stem cells?

Implant dentistry transformed the possibilities of restoration on edentulous patients that had suffered of bone loss. In extreme cases the patients have to go through aggressive surgical procedures in order to compensate severe jaw atrophies, and many times it is not feasible because of the general health of elderly patients. Therefore, gene therapy is presented as an alternative treatment for these patients. Authors indicate that a possibility could be cytokine stimulation of stem cells. However, the question of whether it is effective to stimulate adult stem cells found in these patients, especially in the elderly, is a controversial matter (48).

There is evidence that indicates that aging of the individual, together with that of their stem cells´ lineages do not respond to the induction of cytokines such as BMP as well as it would be desired. Nevertheless, these hypotheses are supported by studies on tissue culture and small animal researches; it would be too precipitated to extrapolate these findings to our patients (32). Indeed, works on primates, indicate that despite a loss in the number of stem cells with aging, using BMP as surgically implanted material seems to promote bone regeneration in large mandibular defects. In elderly hominids Macaca fascicularis, over 25 years of age, a consistent regeneration was proved (49). This is important for the implementation of these cellular therapies in our patients, since the majority of patients for reconstruction of the alveolar bone are in the older age groups. Count of stem cells in the bone marrow and periosteum in patients of 75 years of age, for example, is valued at approximately 1 of every 300,000 cells. This decrease in the number of cells must be taken into account to produce a minimum of response to stimulation with BMP.

What challenges do we have to face in order to use the therapy based on the research on stem cell?

Biological challenges

Despite the evidence showing that regeneration can be achieved in our patients, the difficult goal of clinically achieving a predictable and complete regeneration (as occurs in the case of severe periodontal defects problems) still remains. The isolation and characterization of stem cells obtained from periodontal tissues has provided backing for the understanding of the role of stem cells in wound healing of oral tissues.

However, there are many points that we do not understand at all (27,35). This happens for example in terms of the understanding of signaling mechanisms that dictate aspects as they develop of the tooth root. And beyond, basic aspects of the behavior of stem cells, the molecular basis that dictate the routes which take in their process of self-renewal and differentiation are also largely unknown (50). Regarding that regeneration in oral tissues does happen, we have to repeat the key cellular events that are parallel to the development of such structures, and get the different cell types specifically, apply the same inductive factors and cellular processes involved in the formation of the oral structures to regenerate.

Considering that the final destination of stem cells is influenced by their interaction with their biological niche, in other words, with the microenvironment that surrounds them (including soluble and immobilized factors, extracellular matrix, and signals from neighboring cells), the understanding of these key components of the regulation of stem cells´ properties can elucidate ways to expand stem cells correctly and control their differentiation with precision.

Certainly, numerous studies with stem cells either mentioned or not in this article, have come up to date based on the experimental work in cell culture or animal models. Regarding animal models, it is essential to understand that the results these studies provide have a limited validity when it comes to drawing conclusions for the treatment of our patients. Of course, some species of animals such as primates are best suited for studies than others, as for example mice, and obviously the first help us more to understand the behavior of stem cells in humans (27). With this in mind, even though being essential for a better understanding on this complex field, it is clear that the results of those articles cannot be extrapolated directly to humans.

Technical challenges

The challenges that still need to be solved are related to cell manipulation, biomaterials that serve as support and conduction systems of these components (35). First, the culture conditions have not been sufficiently developed as to imitate the biological niche that surrounds the stem in cells in vivo, and also to ensure that both cell proliferation and differentiation can occur in a safe and consistent way. On the other hand, as the cell culture medium often requires products group (such as fetal bovine serum or mouse feeder layers), cultured cells may not be completely free of pathogens, with the possible risk of infection. Therefore, the establishment of optimal culture conditions without a potential cross-contamination is essential in the production of human stem cells for clinical use and the development of basic research involving the regulation of their self-renewal and the determination of lineage (51).

Secondly, time is an inherent limitation in cell therapy and tissue engineering. Some autologous designs can involve weeks or months of ex vivo processing. Although the use of stem cells can often minimize processing time compared with somatic cells, the potential instability, karyotype and genetic mutations of cells after a long cultivation media can also limit their usefulness. Moreover, the search for the ideal biomaterials support and the system of driving stem cells is an important technical factor. The ideal material that serves as scaffolding must imitate the extracellular matrix that surrounds the cell, serve as support to the union of materials, allow the controlled release of bioactive factors, be conducive for the growth of the tissue and facilitate the manipulation in laboratory (52). Therefore, improvements in the methodology are necessary to incorporate the stem cells to the defects on the oral structures, along with the goal of minimizing the ex vivo processing time and promote the integration and functioning of the implanted cells with cells belonging to the patient.

-Clinical challenges 

The clinical challenges facing stem cells in oral regenerative therapies are related to a possible immune rejection after their administration, oncogenic properties of stem cells and the integration of tissue transplanted to the patient (51). It is important to understand how the immune system can respond to a transplant of stem cells or their derivatives. In general, the immunogenicity of a human cell depends on its expression of the major histocompatibility complex antigen class I and II, allowing the body to differentiate their own cells from foreign cells. Stem cells from human embryos express a low level of major histocompatibility complex antigens class I, but this expression is regulated to differentiation.

A potential solution to this problem could be in the use of autologous stem cells (from cells or tissue banks) to overcome immune rejection. Recently, the research of S. Yamanaka led to the production of patient-specific IPS cells, also known as induced pluripotent stem cells that are extracted from adult somatic cells. However, differentiation of IPS into certain cell types for transplantation is still being studied. The recent findings related to immunosuppression of both *in vitro* and *in vivo* mesenchymal stem cells have also raised the possibility using allogeneic stem cells without the need of a donor and receptor (27).

The challenge in relation to genomic stability and risk of tumor transformation after a stem cell transplant are major concerns in terms of safety, as there are no reliable methods to eliminate undifferentiated embryonic stem cells from the culture medium, and current studies lack long-term follow-up to draw firm conclusions yet. Most probably, the more specific the therapeutic application is, the longer should stem cells remain *in vitro*. During this long period in culture media, it is more probable that genetic or epigenetic changes in stem cells take place. Therefore, a better understanding of such *in vitro* changes will enable a better perception of the risk of tumor genesis in stem cell therapy (35).

Finally, it is not clear whether human stem cells can be integrated and able to perform the specific functions of lost or damaged tissues. It will be necessary to confirm that stem cells are transformed into stable cells showing the characteristics and functions of normal host cells after their transplantation. Hopefully, as a result of the advancement of our knowledge about stem cells, growth factors and cells support systems, a safe and effective cell therapy is expected in the near future.

-Other difficulties

According to Louis Lin *et cols.* in a review of the literature published on periapical surgery and guided tissue regeneration (34,54), it is not possible to correctly perform a meta-analysis in this area due to the wide variations in the methodology of the research. This happens in different types of therapies that are mentioned in the text, focusing especially on stem cells.

In a meta-analysis performed in relation to the possible application of stem cells in sinus floor elevation surgeries (9), authors describe it as an area with deficient quality of studies. An example is that they did not find published randomized clinical trials in patients being studied for both sinuses. In addition, reported studies showed test groups and without any kind of control group. Most studies were performed on a small number of patients and still have a short follow-up (38).

Which conditions should the microenvironment surrounding stem cells ideally have so that they perform their activity in an effective and safe way for the patient?

To apply the therapy based on cells in the treatment of diseases, it is necessary to identify the properties that the microenvironment surrounding them must own (55). It is well known that cell niche highly influences on the behavior of stem cells, thus the appropriate microenvironment provides their optimal performance. These qualities are summarized in six points as Reddi *et al.* (56) propose.

1. The microenvironment surrounding stem cells must increase their survival cells in the receiving area.

2. It must be capable of integrating the transplanted cells without causing damage to the receiver. We need to find strategies to prevent the problem of immune rejection without using immunosuppressive medication.

3. Increase the proliferative activity of stem cells to generate enough tissue.

4. Induce the differentiation of stem cells in the desired cell types.

5. Capable of preserving the cells in that tissue and to continue their activities throughout the life of the patient subject to transplantation.

6. Should identify the unwanted cells and be able to eliminate them.

What future does cell therapy offer? 

Most articles agree to assert that the future of stem cell therapy is promising. However, as occurs with other new technologies, there are many issues that we have to solve. It is now time to move from studies on animals, which are now offering evidence of sufficient safety, to conduct clinical trials of cell therapy on patients. So far, studies on patients are scarce and in few groups of individuals; not enough to obtain statistically significant data. The considerable amount of studies carried out on animals provides an overwhelming amount of evidence that supports the use of mesenchymal stem cells in surgical procedures as in the case of periodontal regeneration.

However, there are critical steps in the establishment of these treatments to for our patients. Problems mentioned that still need to be solved, mainly related to the suitable carriers for them, are: immunogenicity, allogeneic or autologous stem cell source, along with choosing the best donor region, and the control of the global process, without forgetting the cost-benefit ratio. Therefore, before moving forward with confidence, we must pass through a critical phase to validate a systematic treatment with mesenchymal stem cells as a reliable source for therapeutic use.

Finally, dentists interested in the application of cell therapy to their surgical practice would demand the existence of protocols as well as training in this field. Undoubtedly, in the near future there will be an absolute necessity of the development of the infrastructures to process this technology with strict protocols of good manufacturing procedures.

## Conclusions

Research conducted in the field of stem cells has been a revolution in the international scientific community, as an opportunity in dentistry regarding the need of new regenerative technologies. The current regenerative treatments do not provide desirable results; require long periods of pause, and a lack of good clinical predictability.

Hopefully, mesenchymal stem cells are the flagship of the regeneration therapies of tissues. The opportunity of using stem cells has been brought to oral surgery with the discovery of different sources of adult stem cells in oral tissues, as the periodontal ligament or the dental pulp, with the advantage of being easily accessible to dentists.

In various clinical applications mesenchymal stem cells have been used in combination with different matrix and growth factors, showing ability to form periodontal ligament, alveolar bone, cartilage and cement *in vivo*.

Before becoming a realistic alternative in oral tissue regeneration, a thorough understanding concerning self-renewal and differentiation is essential, to overcome the biological, clinical and technical challenges, and thereby facilitate the manipulation of stem cells and their transfer to a clinical environment.

Taking into account that most of the investigations have been limited to the experimental field on animal models, optimistic results must be extrapolated with caution to the clinical application on humans. In our field we still lack of controlled clinical trials with a high level of evidence, so in the next few years there will be a process of trial and error before achieving the clinical protocols of the best materials and combinations.
